# The role of the Mediterranean diet in hyperuricemia and gout

**DOI:** 10.31138/mjr.29.1.21

**Published:** 2018-03-19

**Authors:** Julie Stamostergiou, Xenophon Theodoridis, Vasiliki Ganochoriti, Dimitrios P. Bogdanos, Lazaros I. Sakkas

**Affiliations:** University General Hospital of Larissa, Department of Rheumatology and Clinical Immunology, Faculty of Medicine, School of Health Sciences, University of Thessaly, Larissa, Greece

**Keywords:** diet, hyperuricemia, gout, nutrition, Mediterranean diet

## Abstract

The effect of diet habits in the induction of hyperuricemia and gout is extensively studied and several nutritional factors exacerbating the disease have been identified. In this review, we discuss the data so far obtained of the beneficial role on controlling hyperuricemia of Mediterranean diet, which is full of mono-unsaturated fatty acids and flavonoids and sort of butter, processed food and red meat. We emphasize that though the published findings are promising the data are limited and more studies are needed.

## INTRODUCTION

**Figure 1.**
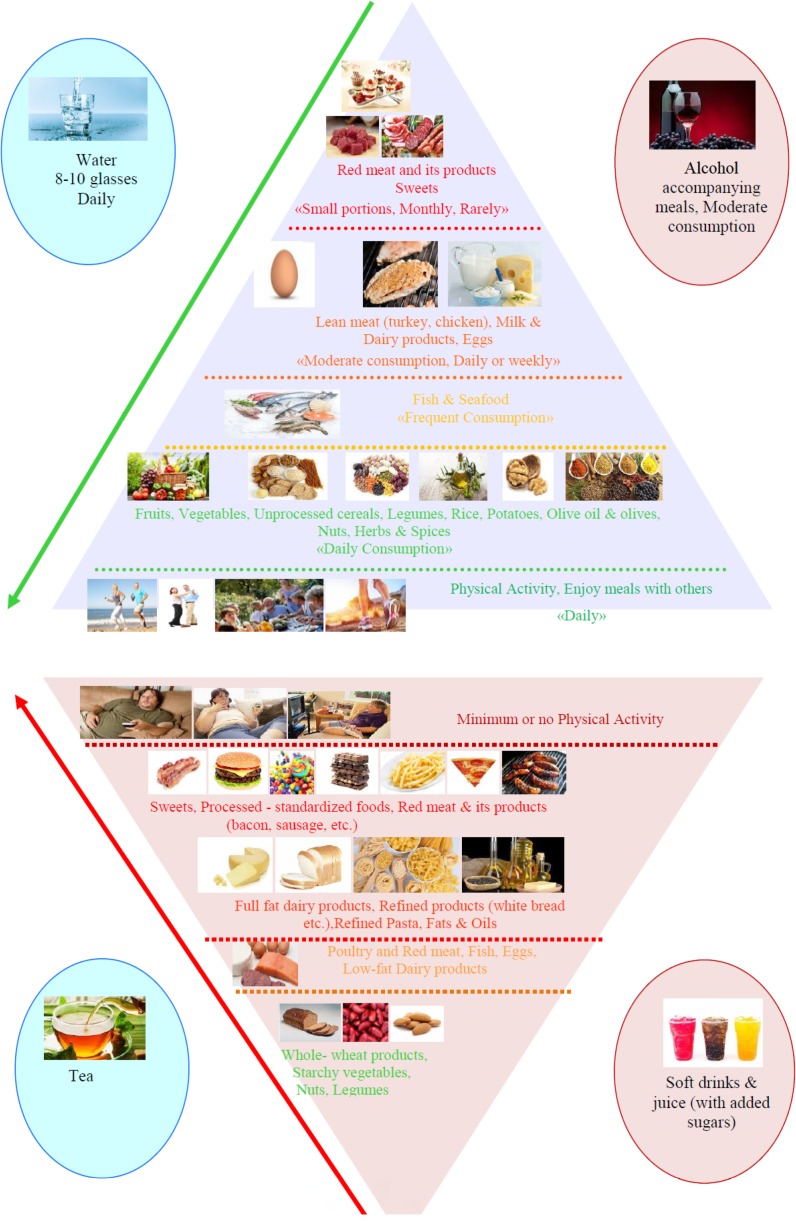
Depiction of a nutritional pyramid according to the Mediterranean diet and Western-type diet. The pyramid in Figure 1 was remodelled and modified based on: The Traditional Healthy Mediterranean Diet Pyramid (Courtesy Oldways Preservation and Exchange Trust, www.oldwayspt.org.

It is widely recognized that each country has its own cultural identity. There is no doubt that eating habits are a big part of a country’s culture. The term “Mediterranean diet” has been used by many scientists to describe the eating habits of the Mediterranean inhabitants, particularly the inhabitants of Crete and Southern Italy, in the early 1960s.^1^ The traditional Mediterranean diet is a dietary model for many countries with many beneficial effects on health, including longevity^2^ and a lower incidence of cardiovascular diseases.^3–8^ This is why many researchers study the relationship between the eating habits of Mediterranean basin inhabitants and their way of living, as well as the correlation with their longevity. The Harvard School of Public Health and the World Health Organization (WHO) in 1993, in conjunction with Greek scientists, schematically represented the Mediterranean Diet with the Nutritional Pyramid, making it a “standard” that should be followed for lifetime for the preservation and protection of public health. The basis of this pyramid includes foods that should be consumed and habits that should be followed on a daily basis. Going to the top of the pyramid there are foods, that are supposed to be consumed on a weekly or monthly basis (**Figure 1**).

### Characteristics of the Mediterranean diet

The Mediterranean diet focuses on cereals (bread, oats, wholegrain cereals, groats), fruits, vegetables, nuts and legumes, whose consumption should be on a daily basis. These foods are rich in fiber and antioxidants (especially if they are seasonal). Consumption of olive oil replaces other forms of saturated fat, such as animal butter and margarines. In moderate consumption, dairy products, especially yoghurt and cheese, are found even on a daily basis, while consumption of fish and poultry (lean animal products) is recommended up to twice a week. Eggs can be eaten up to 4–7/week. Climbing up the food pyramid, there are foods which should be consumed on a monthly basis in small quantities, such as red meat. Alcohol accompanies each meal and if there is no other problem associated with its consumption then the safe recommended units are 2 glasses/day for men and 1 glass/day for women. There is a preference for red wine because of its flavonoids and antioxidants content. In addition to dietary characteristics, emphasis is also put on moderate daily physical activity to maintain a normal weight, to achieve well-being and to help eliminate diseases that are caused by excess body weight.

Many Mediterranean countries have common components with this Mediterranean diet model, such as low consumption of red meat and its replacement by fish or other protein sources such as legumes. But what makes it special in terms of its characteristics and is considered by many countries as an optimum dietary model? Through many large studies, it seems that the main features that offer many beneficial effects on the health of these people are two: Mono-unsaturated fatty acids in olive oil, mainly oleic acid, poly-unsaturated fatty acids in particular n-3 found in oily fish and walnuts,^4^ as well as flavonoids of red wine (accompanying meals) are the main ingredients that combined with all of the above mentioned foods lead to the maximum benefits to the organism’s longevity and consequently to the reduction in overall mortality.^9–10^ Most studies have focused on the role of the Mediterranean diet in reducing the incidence of cardiovascular diseases,^4,11–14^ the prevention of type 2 diabetes^15–17^ and consequently the metabolic syndrome^18^ as well as other pathological health conditions. The Seven Countries Study, led by Ansel Keys and his associates, began in the early 1950s and lasted for several years.^19^ Many people from Greece (Crete and Corfu) participated in this study, as well as people from Yugoslavia, Italy, the Netherlands, Finland, USA and Japan.^19^ The authors concluded that the traditional Mediterranean diet has a number of benefits to our health. Also, the Lyon Heart Study, a randomized study, showed a significant cardioprotective role of the Mediterranean diet.^11–12^ The results of the PREDIMED study advocates to the previous results.^4,16^ A major drawback of these studies is that it is not clarified whether or not other alcoholic drinks are consumed and what their effect may be. In addition, elaic acid, the main ingredient of olive oil, is also found in beef and pork, but its role in these animal sources is not specified either. The surveys were conducted in Mediterranean countries, and the question is whether or not the implementation of Mediterranean diet in other countries in USA or Europe would have the same beneficial effects given the different lifestyle there. In conclusion, it is crucial to have further research on the health effects of the Mediterranean diet in other countries.

**Figure 2.**
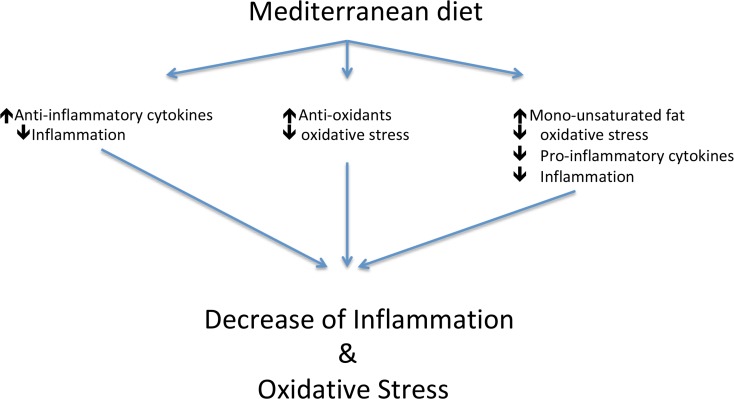
Mediterranean diet exerts its beneficial effect mainly through the reduction of pro-inflammatory cytokines and increase of anti-inflammatory cytokines, as well as, the attenuation of oxidative stress.

The Mediterranean diet is rich in antioxidants and vitamins and has anti-inflammatory properties. Thus, it reduces pro-inflammatory cytokines, increases anti-inflammatory cytokines and reduces oxidative stress (**Figure 2**). Diet per se seems to have little effect on serum uric acid (SUA) levels,^20^ and a diet low in purines can reduce SUA by 10–15%.^21^

Three studies examined the effect of Mediterranean diet on hyperuricemia and hence on gout, the Ikaria study,^22^ the ATTICA study^23^ and the PREDIMED study.^24^ The Ikaria study, conducted in the east Aegean island of Ikaria, Greece, included 281 women and 257 men of at least 65 years of age with no known cardiovascular disease and assessed the effect of Mediterranean diet on uric acid.^22^ The tools used in this study were a food frequency questionnaire (FFQ) and a physical activity questionnaire (IPAQ).The food frequency questionnaire consisted of food groups and drinks consumed on a daily or weekly basis. Examples include red meat and meat products, fish and seafood, poultry, milk and other dairy products, fruits, vegetables, legumes, unprocessed cereals, coffee, tea, soft drinks and alcohol. It should be noted that unit of measurement was a wineglass in ml (1 wineglass is equivalent to 12 g of ethanol). In addition to food, nutritional assessment was done using the MedDietScoreindex.^25^ This index has a score ranging from 0 to 55. The highest score the greater the adherence to Mediterranean diet. The results of this study pointed out that SUA levels decrease independently of other measurements (creatinine, body mass index and other clinical characteristics), even with a small increase in the MedDietScore, although this was not clearly evident for women.

The ATTICA study, conducted in Greece, included 2380 men and women without cardiovascular (CHD and CVD) or renal disease (CKD). Individuals on drugs that may have affected SUA were excluded.^23^ Cardiologists, nutritionists and nurses used a quality of life questionnaire and performed biochemical and clinical measurements. They also incorporated 156 foods and beverages consumed in Greece in a food and beverage consumption questionnaire tool, used by nutritionists. The nutritional assessment was carried out with the use of MedDietScore. Score 0 was assigned to the consumption of foods that are away of the Mediterranean pattern, while scores 1 to 5 were assigned to each question when participants reported rare to daily consumption of foods following the Mediterranean pattern. The nine food groups evaluated in the MedDietScore were: whole grain cereals, fruits, vegetables, legumes, potatoes, fish, red meat and its products, poultry and whole fat dairy products (cheese, yogurt etc.). It also included the daily use of olive oil in cooking as well as the daily alcohol consumption.^25^ In accordance to the NHANES study,^21^ they found that dairy products seem to reduce SUA levels, while there was a positive correlation between coffee and alcohol consumption and SUA levels. Greater compliance with the Mediterranean diet resulted in a better outcome on SUA.^23^

**Figure 3.**
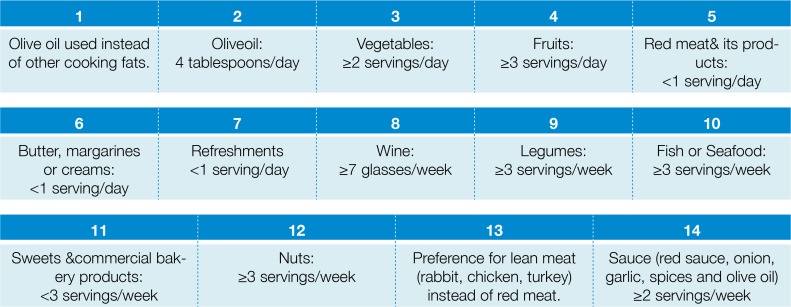
Dietary factors studied in PREDIMED.

The PREDIMED^24^ study, conducted in Spain, included 4449 individuals aged 55 to 80 years and looked at 14 dietary factors (**Figure 3**) and examined the effect of dietary compliance to SUA. It lasted for five years. They included individuals with type 2 diabetes, or with three or more criteria for coronary heart disease. These are similar to those of metabolic syndrome: hypertension 140/90mmHg or use of antihypertensive drugs, HDL ≤40mg/dl for men and HDL≤ 50mg/dl for women, LDL≥150mg/dl, BMI≥25 kg/m^2^ (overweight or obese patients), a family history of cardiovascular disease at age ≤55 years old for men and ≤60 years old for women respectively and smoking. Exclusion criteria were BMI ≥40 kg/m^2^, chronic health conditions, use of alcohol or drugs, allergy or intolerance to olives, olive oil or nuts. Three dietary patterns were tested and had the same effect. In the first group they applied a Mediterranean diet enriched with extra virgin olive oil (MEDIET + EVOO). In the second group they used the Mediterranean diet enriched with nuts (MEDIET + NUTS). The third group used a low-fat control diet. The results of the study showed that both men and women of all ages adhering to Mediterranean diet reduced SUA levels. Approximately 44% (43.8%) of individuals managed to reduce SUA levels by following the Mediterranean diet.^24^ Moreover, similar results have emerged for overweight people, hypertensive, smokers and non-smokers, but also for individuals with and without physical activity. The results of the study were as follows: frequent consumption of large quantities of meat and fish (mainly seafood) resulted in an increase in SUA levels, whereas reduced frequency and quantity of those led to a decrease in SUA levels. The same results emerged concerning the frequency and quantity of alcoholic beverage consumption.^24^ Overconsumption of beer and other alcoholic beverages seems to increase the risk of gout. On the contrary, moderate wine consumption (<7 glasses of wine/week), due to its polyphenols content (compounds with antioxidant properties), does not seem to act as a factor influencing the prevalence. Also, legumes and “sofrito” sauce appeared to have a positive effect. Legumes are rich in fiber and protein, while “sofrito” sauce stands for tomato sauce with many spices and ingredients with plenty of antioxidant and anti-inflammatory properties, which help reduce inflammation. However, ¼ of the individuals without hyperuricemia at the onset of the study, developed hyperuricemia despite their adherence to the Mediterranean diet.^24^

In conclusion, the results of these studies on Mediterranean diet are encouraging. However, there is a need for more studies in gout to see if Mediterranean diet in combination with other non-nutritional parameters may prevent gout or reduce gouty attacks.
